# Institutional Housekeeping

**Published:** 1912-11-09

**Authors:** 


### INSTITUTIONAL HOUSEKEEPING.

### The Domestic School—Housemaids.

EVERYTHING that is done perfectly becomes an art. "Blessed be drudgery," it has been said, but its chief blessing is only known when it has changed its name. There is no drudgery in maintaining a high standard even where cleanliness and order are the simple objects of our ambition. A whole army of housemaids cannot make a family or an establishment comfortable if their work is done perfunctorily and they are only careful when overlooked. They are a worry and necessary nuisance to everyone. But let the matron instil into a fair number of young women the principle of responsibility, the pride of reliability, and the delight of earning credit for thoroughness, and the housework becomes almost as inspiring and interesting as the nursing. Girls of sixteen have to be trained in standing alone. We want to create a public spirit in our household quite as much as in our wards. It is difficult to do, and it takes a long time in forming, but when the public spirit is well stamped in it sticks for years and saves a world of trouble. The domestics are not all changed at the same time, and sufficient remain to carry on the credit of the establishment and show newcomers what is expected of them. "Oh, we never do that here!" says a housemaid to a new arrival, in a shocked voice, and the others look horrified till the new girl wishes she hadn't. Dormitory work can be made actually agreeable if a legitimate pride and pleasure is inculcated into each individual worker. Every duty should be carefully and plainly written down; and the housemaids who get through their work well and punctually soon understand they are on the way to promotion, and will respond if entrusted now and then with higher duties. There are always some things too important to be left to novices who are certain to be careless now and then. A moment's neglect in allowing matches or hair to pass into the slop-pail may occasion whole weeks of trouble with the drainage. Housemaids should be taught to think of these things, and encouraged to feel responsibility for the establishment as when they run upstairs in a thunderstorm to shut the windows without any reminder, or draw attention to some curtain dangerously near a gas light. We must expect in young servants many slovenly and often very horrid tricks and be on the watch for them. They do not see the reason for scrupulous cleanliness in the bedrooms nor for putting fresh water in the jugs and water-bottles every day, and will leave it for a week if no one is on guard. Then, of course, we know many will open drawers and boxes, read letters, and do anything short of stealing, which is a rare default happily. All these things are natural to untaught girls—mere tricks and habits, not crimes for which the poor things must be dismissed peremptorily. If the domestic workers be secured at an impressionable age all these vagaries will soon be things of the past. A cheerful little band of willing workers, if trained under a person who is herself well-trained, will grow to look upon their vocation as not the least honourable in the establishment. But from the first a high standard must be set. No pains should be spared to show young servants how to do their work daintily and with finish, and to convince them it is to their interest to be particular by ensuring steady promotion.

### Readers' Questions with Practical Answers.

**Cheap Spoons and Forks.**—We advise you not to embark in very low-priced electroplate. The silver coating soon wears off under hard use. You can get Nevada nickel-silver at about half the cost of electroplate, and it will wear white to the end, and take a good polish.—ESTHER B.

**Prune Stone Cream.**—It is quite true that a great many people dislike stewed prunes, and the difficulty is to find an adequate substitute during the winter months, but the following way of cooking prunes makes a pleasant change: Soak half a pound of prunes overnight in just sufficient water to cover them, stew the prunes in the water they were soaked in with two or three lumps of sugar; to give them a good colour you may use a little carmine, but this is not necessary. When soft, remove the stones; place the prunes in a flat dish when cool, and smooth them flat; boil one pint of milk with one or two almonds, sweeten to taste, add a few leaves of gelatine, and when nearly cold pour it over the prunes very slowly. If desirous of making it look more decorative blanch and strip 1 oz. of almonds and stick all over.—HOUSEKEEPER.

